# Urban–Rural Differences in Mental and Physical Health among Primary Care Patients with Multiple Chronic Conditions: A Secondary Analysis from a Randomized Clinical Trial

**DOI:** 10.3390/ijerph192315580

**Published:** 2022-11-24

**Authors:** Levi N. Bonnell, Jessica Clifton, Gail L. Rose, Elizabeth N. Waddell, Benjamin Littenberg

**Affiliations:** 1 Department of Medicine, University of Vermont, Burlington, VT 05405, USA; 2 Department of Psychiatry, University of Vermont, Burlington, VT 05405, USA; 3 Division of General Internal Medicine and Geriatrics, Oregon Health and Science University, Portland, OR 97201, USA

**Keywords:** rural health, mental health, physical health

## Abstract

Purpose: Rural health disparities are largely attributable to access to healthcare, socioeconomic status, and health behaviors. Little is known about the persistence of these disparities when differences in access to care are eliminated. We sought to investigate urban–rural differences in physical and mental health in primary care patients with demonstrated access to primary care. Methods: We obtained cross-sectional survey responses from a multicenter randomized controlled trial on 2726 adult primary care patients with multiple chronic medical or behavioral conditions from 42 primary care practices in 13 states. Study outcomes include measures of mental health including: The Patient-Reported Outcomes Measurement Information System (PROMIS-29^®^), General Anxiety Disorder-7 (GAD-7), and Patient Health Questionnaire-9 (PHQ-9), as well as physical health including: the PROMIS-29^®^ and the Duke Activity Status Index (DASI). Urban–rural residence was indicated by census-tract Rural Urban Commuting Areas of the participant’s home address. Differences in mental and physical health outcomes attributable to rurality were assessed using multilevel models with a random intercept for census-tract. Results: After adjustment for demographic and neighborhood characteristics, urban residents had significantly worse generalized anxiety disorder (GAD-7) (ß = 0.7; 95% CI = 0.1, 1.3; *p* = 0.027), depression (PHQ-9) (ß = 0.7; 95% CI = 0.1, 1.4; *p* = 0.024), and functional capacity (DASI) (ß = −0.4; 95% CI = −0.5, −0.2; *p* < 0.001) compared to rural residents. Urban residents also had significantly worse anxiety and depression as measured by the PROMIS-29^®^ compared to their rural counterparts. There were no urban–rural differences in the other PROMIS-29^®^ subdomains. Conclusions: Among adults with demonstrated access to care and multiple diagnosed chronic conditions, rural residents had better mental health and functional capacity than their urban counterparts. This finding is not consistent with prior research documenting rural health disparities and should be confirmed.

## 1. Introduction

Prior research clearly documents striking disparities in health status between rural and urban Americans. Access to care, which is typically worse in rural areas, is associated with lower rates of all-cause mortality [1]. Rural Americans are more likely to report risk factors associated with poorer health including poverty [2], food and housing insecurities [3,4,5], tobacco and substance misuse [6,7,8], and physical inactivity [9,10]. Moreover, rural Americans face unique barriers to accessing and utilizing healthcare because of limited transportation [11], insurance coverage [12,13], access and utilization of health information [14], broadband internet services [15], and healthcare facilities and workforce [16,17].

There is a widening gap in mortality rates, with rural communities experiencing a higher proportion of excess deaths from heart disease, cancer, unintentional injury, chronic lower respiratory disease, and stroke [18]. Many of these conditions co-occur with other medical and behavioral conditions. Most US studies documenting urban–rural disparities have found that rural residents suffer poorer health across a variety of outcomes. A recent meta-analysis found that the prevalence rates of mental health disorders were higher in rural than urban settings [19]. Among a large sample of US Veterans with comprehensive healthcare benefits, rural residents had worse mental and physical health [20]. Similarly, using the National Health Interview Survey, Probst et al. revealed a higher prevalence of depression among rural US residents [21]. A study based on the US National Survey of American Life found that rural African American women had overall worse health than their urban counterparts [22]. Additionally, rural residents in North Carolina were more likely to have chronic pain than their urban counterparts [23].

The mechanisms that drive urban–rural health disparities are multifactorial and complex. Managing multiple chronic conditions involves more healthcare interaction. Lack of access to care in rural areas presents enormous barriers to management of chronic conditions requiring more office visits to maintain function and prevent worsening. To assess whether effective access to care is responsible, we investigated urban–rural differences in mental, physical, and social health and well-being among primary care patients with demonstrated access to and utilization of primary care services. We hypothesized that rural residents with access to care would have similar health outcomes as their urban counterparts with similar access to care.

By assessing disparities in outcomes within a geographically diverse sample of high-risk patients who are engaged in primary care, we are able to explore differences that are not directly associated with access to and utilization of healthcare or health insurance. Note that this study took place before the SARS-CoV-2 global pandemic hit in early 2020.

## 2. Materials and Methods

### 2.1. Data and Setting

Baseline survey results (pre-COVID) were obtained from Integrating Behavioral Health and Primary Care, a multi-center randomized study of primary care patients from 2016–2021, described elsewhere [24]. Data were collected from adults with multiple chronic medical and behavioral conditions (at least two of heart disease, diabetes, lung disease, arthritis, mood disorder, insomnia, substance abuse, chronic pain, or irritable bowel syndrome) from 44 primary care practices across 13 states (Alaska, Hawaii, Washington, Oregon, California, Idaho, Texas, Georgia, Kentucky, Ohio, New York, Massachusetts, Vermont) with co-located behavioral health services. Practices were selected via convenience sample, but they represent all the regions of the US across various social, built, and natural geographies. All patients had evidenced access to and utilization of primary care, as demonstrated by at least two primary care visits over 24 months for any purpose, including at least one in the most recent six months. Patients under 18 years old were excluded. Chronic conditions and visit history were identified through review of their electronic medical record. There were 4025 records collected from patients. Many of these were incomplete or not eligible for the study. 3006 respondents met the inclusion criteria for the main study and 2726 had complete data for the predictor and outcomes for this sub-analysis. Using this unique dataset granted us the opportunity to assess how residing in rural areas affected health independent of access to and utilization of care.

The outcomes were mental and physical health as measured by the Patient-Reported Outcomes Measurement Information System^®^-29 (PROMIS-29) [25,26], Generalized Anxiety Disorder-7 (GAD-7) [27], Patient Health Questionnaire-9 (PHQ-9) [28], and Duke Activity Status Index [29] (DASI). The PROMIS-29 is a self-reported questionnaire that assesses eight domains of health including pain interference, pain intensity, physical function, depression, anxiety, fatigue, sleep disturbance, and social participation. From these domains, physical and mental health summary scores are calculated. Except for pain intensity, which is reported on a 0–10 scale, PROMIS^®^-29 scores are reported in t-scores such that the mean of the adults US population is 50 and the standard deviation is 10. Higher scores indicate better function for the physical and mental health summary scores, and the physical function and social participation domain scores, while lower scores indicate better outcomes for pain interference, pain intensity, depression, anxiety, fatigue, and sleep disturbance. For the parent clinical trial, a change in PROMIS-29 of ~5 was considered to be a minimally clinical important difference (MCID).

The GAD-7 and PHQ-9 are brief questionnaires that measure generalized anxiety disorder and depression symptom severity on continuous scales from 0–21 and 0–27, respectively. Although the GAD-7 and PHQ-9 are sometimes reduced to categories for ease of interpretation, we report them in their original continuous scoring to preserve the greatest amount of information. Higher scores indicate increased severity. The authors report that a MCID is between 3 and 5 [4,5]. The DASI is a 12-item questionnaire that assess the ability to do self-care, housework, sports, and other activities as well as estimates maximal oxygen consumption [30,31]. For this analysis, we converted the DASI to Metabolic Equivalent of Task (METs) units (2.74–9.89) where higher METs indicate better functional capacity. For this analysis, all outcomes were measured continuously. Although a MCID is not reported by the authors, the DASI can be converted to METs, where roughly 0.75 change corresponds to 10% of total functional capacity. This is roughly equivalent to being able to walk briskly (3.4 MPH) versus walking at a leisurely pace (2.5 MPH).

The primary predictor was urban vs. rural defined by Rural Urban Commuting Areas (RUCA), a census tract classification scheme based on population density, urbanization, and daily commuting patterns [32]. The urban category includes secondary RUCA values of 1.0, 1.1, 2.0, 2.1, 4.1, 5.1, 7.1, 8.1, and 10.1; all others were rural. The distribution of participants categorized as urban vs. rural by the original RUCA codes are displayed in Table 1. The majority of rural participants resided in RUCA code 10 followed by 4 and 7. Although there are many descriptive definitions of rurality, no single measure is universally appropriate. The RUCA codes combine US Census Bureau classifications [33] with data on the commuting habits of residents in each census tract [32].

Potential covariates were chosen based on clinical knowledge and prior literature on urban–rural differences in health. Individual and neighborhood-level covariates considered were age, gender (male vs. female), race (white, black or African American, Asian, Other), ethnicity (Hispanic vs. Non-Hispanic), marital status (married or living-as-married vs. not), employment status (employed defined as full-time, part-time, student or homemaker vs. not), annual household income household income (<$30,000 vs. ≥$30,000), education (high school graduate or less vs. associates degree or more), the number of qualifying chronic conditions, and the Social Deprivation Index [34] (SDI). SDI is a census tract-level composite measure of deprivation derived from the American Community Survey based on income, education, employment, housing, single-parent household, and access to transportation.

### 2.2. Statistical Analysis

We used Wilcoxon rank-sum tests to compare physical and mental health between those residing in rural vs. urban areas. Relative differences of health outcomes between urban and rural populations were calculated. Several multilevel linear regression models were used to estimate the mean difference in each outcome by urban–rural status with its 95% confidence interval (CI). Census tract was included as a random intercept to account for correlation of patient-level measures within their home neighborhood. All predictors and covariates were included as fixed effects.

If a covariate changed the coefficient of urban vs. rural on the outcome by more than ±10% in a model with no other covariates, it was included in the final model as a potential confounder. Covariates were assessed for collinearity before inclusion in the multivariable model. All tests were two-tailed and the threshold for statistical significance for the main analysis was set at α = 0.05. Stata 16.1 (StataCorp LP, College Station, TX, USA) was used for data management and statistical analysis. The University of Vermont Institutional Review Board approved this study.

## 3. Results

The average age of participants was 62 years with an interquartile range of 53–71 years. As expected in a population with multiple chronic conditions, all PROMIS^®^-29 scores except the mental health summary score were below the average US population score of 50. Rural residents comprised 19% of the sample, a similar proportion to the US population. Participants residing in rural areas were more likely to be married, white, non-Hispanic, older, finish education without a college degree, and have fewer chronic conditions. Notably, each measured aspect of health was worse among patients living in urban areas compared to their peers living in rural areas who utilized similar care (Table 2). Residing in an urban area was associated with a relative change of 6% higher generalized anxiety disorder symptom severity, 5% lower total functional capacity, and 4% more depression symptom severity.

**Figure 1:**
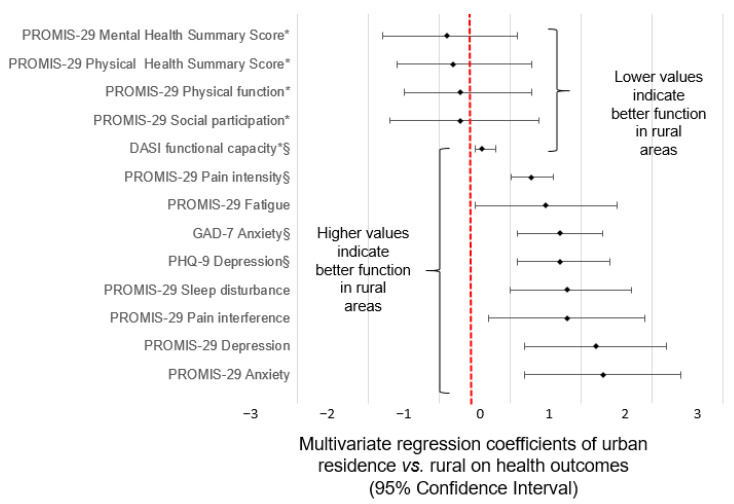
Urban vs. rural differences in mental, physical, and social health and well-being. Regression coefficient (β) and 95% Confidence Interval for the mixed linear regression with adjustment for all potential confounders. The vertical line at zero represents a null association. The regression coefficients indicate the scores of urban residents relative to rural residents. In each case, the effect suggests better outcomes among rural residents, although not all are statistically significant. * Reverse scale: lower scores are worse ^§^ Different scale.

After adjustment for relevant potentially confounding factors, a greater proportion of urban residents reported having generalized anxiety disorder and depression, and worse functional capacity than their rural counterparts. Although physical function, social participation, chronic pain severity, pain intensity, sleep, and fatigue lost statistical significance in the multivariable analysis, all of the coefficients remained consistent with a greater proportion of urban residents reporting having poorer health (Table 3 and Figure 1).

There was 3% missing data in the final multilevel models. Participants with missing data did not significantly differ by age, sex, race, ethnicity, or urban residence from those without missing data.

## 4. Discussion

We explored urban–rural differences in patient-reported health outcomes among US adults with multiple chronic conditions and demonstrated access to and utilization of primary care services. This unique study population allowed for investigation of rural disparities not directly attributable to the reduced access to and utilization care characteristic of rural areas. Among a highly vulnerable sample of patients from urban and rural primary care practices with co-located behavioral health services, urban residents had significantly worse generalized anxiety disorder, depression, and functional capacity as compared to their rural counterparts with similar utilization of care, and had non-significantly worse scores on all other health outcomes measured. These findings stand in contrast to multiple US studies that have shown urban residence is associated with better health [19,20,21].

Although the observed differences in outcomes between urban and rural populations were smaller than the MCIDs, they were statistically significant. MCIDs are intended to assess the clinical impact of interventions not the public health significance of environmental factors [35]. Although these associations are small, they still highlight potentially meaningful differences at a population level.

A unique characteristic of the current study is that all participants had demonstrated access to primary care services, a factor that is associated with less financial burden, fewer disparities, and better health outcomes [36]. Primary care can prevent illness by promoting healthful lifestyles and identifying and treating patients that have risk factors for disease but have no symptoms. Further, primary care and behavioral health services help manage chronic conditions and prevent them from progressing, an especially important need for the participants of this study who all had multiple chronic conditions. However, rural US residents have barriers accessing primary and specialty care such as increased travel distances. Our results suggest that access to primary care services may at least partially underlie the discrepancies in health outcomes observed in other studies of urban and rural health.

There are important limitations to consider. First, the data were collected in 13 states and may not be generalizable to the US population. However, we have a representation of urban and rural residents from all 13 states where we collected data. Further, it is unclear if these results will apply to other forms of primary care, as all practices had a co-located behavioral health provider. Second, this study was cross-sectional; causal inference was not possible. Third, although we controlled for several potential confounding factors, we cannot eliminate the possibility of unmeasured confounders. In particular, we did not have data on health insurance. However, all participants had received care in the previous six months, indicating good access. Fourth, although the urban–rural distribution is representative of the US population, there are still many more urban participants than rural, which lead to small samples sizes and low statistical power for some outcomes. Fifth, reporting behavior could be intrinsically different between residents residing in urban and rural areas. For instance, we cannot identify if rural residents are more or less likely to recognize or report mental health symptoms compared to their urban counterparts. Finally, we chose RUCA because it is commonly used in health services research and more precise than county and zip code measures, but results might differ if alternative classifications were used.

This analysis of urban–rural differences in mental and physical health is the first to use a sample of US residents with multiple chronic conditions, demonstrated access to healthcare, and the first study anywhere to assess urban vs. rural differences in functional capacity. Our findings suggest that access to primary care may be an important factor in explaining the gap in urban–rural health disparities, and suggest that improving rural access may warrant investigation as a means to reduce those disparities. Because this is the first study to have examined urban–rural health status among a sample who all could access and utilize health care, more studies are needed to better understand the relationship between access and health status.

## Figures and Tables

**Table 1:** Distribution of participants categorized as urban vs. rural across secondary RUCA codes, 2010.

	Rural	Urban
	N = 518	N = 2208
* Secondary RUCA Codes—Urban		
1.0	0 (0%)	1965 (89%)
1.1	0 (0%)	53 (2%)
2.0	0 (0%)	135 (6%)
2.1	0 (0%)	5 (0%)
4.1	0 (0%)	1 (0%)
5.1	0 (0%)	1 (0%)
7.1	0 (0%)	48 (2%)
* Secondary RUCA Codes—Rural		
3.0	3 (1%)	0 (0%)
4.0	140 (27%)	0 (0%)
5.0	36 (7%)	0 (0%)
6.0	2 (0%)	0 (0%)
7.0	62 (12%)	0 (0%)
8.0	33 (6%)	0 (0%)
10.0	237 (46%)	0 (0%)
10.2	5 (1%)	0 (0%)

* Secondary RUCA categories with no participants are not displayed in the table including 4.2, 5.2, 6.1, 7.1, 7.2, 7.3, 7.4, 8.1, 8.2, 8.3, 8.4, 9.0, 9.1, 9.2, 10.1, 10.3, 10.4, 10.5, 10.6.

**Table 2:** Characteristics of the participants.

	Overall N (%) or Mean ± SD	Urban N (%) or Mean ± SD	Rural N (%) or Mean ± SD
N	2726	2208 (81%)	518 (19%)
Demographic information			
Mean age ± SD, year	61.7 ± 13.4	61.5 ± 13.4	62.6 ± 13.5
Female gender	1742 (64%)	1404 (63%)	338 (65%)
White race	2052 (77%)	1607 (75%)	445 (87%)
Non-Hispanic White	1926 (71%)	1491 (68%)	435 (84%)
Non-Hispanic Black	299 (11%)	294 (13%)	5 (1%)
Hispanic (any race)	271 (10%)	248 (11%)	23 (4%)
Non-Hispanic (other)	230 (8%)	175 (8%)	55 (11%)
Married or living as married	1309 (48%)	1016 (46%)	293 (57%)
Employed	906 (34%)	722 (33%)	184 (36%)
Low household income ($ < 30 k)	1408 (53%)	1158 (54%)	250 (50%)
Education (Less than a college degree)	1462 (54%)	1158 (53%)	304 (59%)
Mean number of chronic conditions ± SD	4.1 ± 1.8	4.2 ± 1.8	3.9 ± 1.6
Neighborhood characteristics			
Mean social deprivation index	53.5 ± 27.9	53.3 ± 29.9	54.2 ± 16.5
Chronic conditions			
Arthritis	1140 (42%)	947 (43%)	193 (37%)
Asthma	596 (22%)	488 (22%)	108 (21%)
Chronic Obstructive Pulmonary Disease	889 (33%)	719 (33%)	170 (33%)
Chronic pain	2285 (84%)	1866 (85%)	419 (81%)
Non-Gestational Diabetes	1238 (45%)	1052 (48%)	186 (36%)
Heart failure	227 (8%)	196 (9%)	31 (6%)
Hypertension	2244 (82%)	1850 (84%)	394 (76%)
Irritable bowel syndrome	115 (4%)	88 (4%)	27 (5%)
Anxiety or depression	1734 (64%)	1402 (64%)	332 (64%)
Insomnia	673 (25%)	558 (25%)	115 (22%)
Substance use disorder	624 (23%)	514 (23%)	110 (21%)
Outcomes			
Mental health summary score *	50.1 ± 8.9	49.8 ± 8.9	51.5 ± 8.8
Physical health summary score *	45.7 ± 9.6	45.4 ± 9.5	47.0 ± 9.6
Physical function *	43.4 ± 9.5	43.1 ± 9.5	44.6 ± 9.5
Social participation *	48.1 ± 10.1	47.8 ± 10.1	49.3 ± 10.1
DASI (METs) *	6.3 ± 2.0	6.2 ± 1.9	6.8 ± 1.9
Pain intensity	4.5 ± 2.8	4.6 ± 2.8	4.0 ± 2.6
Fatigue	52.7 ± 10.4	52.7 ± 10.5	51.6 ± 10.1
Sleep disturbance	53.2 ± 8.9	53.5 ± 8.9	52.0 ± 8.9
Pain interference	58.3 ± 10.1	58.6 ± 10.1	57.0 ± 10.0
Depression	52.9 ± 9.8	53.3 ± 9.9	51.4 ± 9.4
Anxiety	54.1 ± 10.1	54.5 ± 10.2	52.4 ± 9.8
PHQ-9	6.5 ± 5.4	6.8 ± 6.2	5.5 ± 5.7
PHQ-9 Categories			
None	1311 (49%)	1027 (48%)	284 (56%)
Mild	671 (25%)	546 (25%)	125 (25%)
Moderate	349 (13%)	301 (14%)	48 (9%)
Moderately Severe	202 (8%)	168 (8%)	34 (7%)
Severe	133 (5%)	115 (5%)	18 (3%)
GAD-7	4.6 ± 5.3	4.8 ± 5.4	3.8 ± 4.8
GAD-7 Categories			
Minimal	2267 (85%)	1821 (83%)	446 (88%)
Mild	223 (8%)	189 (9%)	34 (7%)
Moderate	138 (5%)	122 (6%)	16 (3%)
Severe	65 (2%)	55 (2%)	10 (2%)

* Reverse scale: lower scores are worse.

**Table 3:** Unadjusted and multivariable models.

	Unadjusted			Adjusted		
Outcomes	Association of Urban vs. Rural	95% CI	*p*	Association of Urban vs. Rural	95% CI	*p*
^a^ PROMIS-29 Mental health summary score *	−1.5	−2.6, −0.5	0.005	−0.9	−1.8, 0.1	0.071
^b^ PROMIS-29 Physical health summary score *	−1.5	−2.6, −0.4	0.01	−0.8	−1.6, 0.3	0.066
^c^ PROMIS-29 Physical function *	−1.4	−2.5, −0.3	0.01	−0.7	−1.5, 0.2	0.118
^d^ PROMIS-29 Social participation *	−1.3	−2.5, −0.1	0.03	−0.7	−1.7, 0.4	0.225
^d^ DASI functional capacity *	−0.5	−0.7, −0.2	<0.001	−0.4	−0.5, −0.2	<0.001
^e^ PROMIS-29 Pain intensity	0.6	0.2, 0.9	0.004	0.3	−0.0, 0.6	0.067
^f^ PROMIS-29 Fatigue	1.3	0.1, 2.4	0. 03	0.5	−0.5, 1.5	0.308
^g^ GAD-7 Anxiety	0.9	0.2, 1.6	0.008	0.7	0.1, 1.3	0.027
^g^ PHQ-9 Depression	1.1	0.3, 1.8	0.004	0.7	0.1, 1.4	0.024
^h^ PROMIS-29 Sleep disturbance	1.4	0.4, 2.4	0.008	0.8	−0.1, 1.7	0.098
^g^ PROMIS-29 Pain interference	1.4	0.2, 2.7	0.03	0.8	−0.3, 1.9	0.172
^i^ PROMIS-29 Depression	1.8	0.6, 2.9	0.002	1.2	0.2, 2.2	0.016
^j^ PROMIS-29 Anxiety	2.1	0.8, 3.3	0.002	1.3	0.2, 2.4	0.021

^a^ Confounder list: Marital status, education, number of chronic conditions; ^b^ Confounder list: Marital status, employment, income, education, SDI, number of chronic conditions; ^c^ Confounder list: Marital status, employment, income, education, number of chronic conditions; ^d^ Confounder list: Marital status, employment, education, SDI, number of chronic conditions; ^e^ Confounder list: Race, ethnicity, marital status, education, SDI, number of chronic conditions; ^f^ Confounder list: Age, marital status, income, number of chronic conditions; ^g^ Confounder list: Race, marital status, education, SDI, number of chronic conditions; ^h^ Confounder list: Race, marital status, education, number of chronic conditions; ^i^ Confounder list: Marital status, income, number of chronic conditions; ^j^ Confounder list: Marital status, number of chronic conditions; * Higher scores indicate better outcomes.

## Data Availability

The data presented in this study are available on request from the corresponding author. The data are not publicly available yet because data are still being cleaned.
